# Myocardial lipin1 protects the heart against ischemic injury by preserving lipid homeostasis

**DOI:** 10.1172/jci.insight.183334

**Published:** 2025-10-30

**Authors:** Jiaxi Guo, Kohei Karasaki, Kazutaka Ueda, Manami Katoh, Masaki Hashimoto, Toshiyuki Ko, Masato Ishizuka, Satoshi Bujo, Chunxia Zhao, Risa Kishikawa, Haruka Yanagisawa-Murakami, Hiroyuki Sowa, Bowen Zhai, Mutsuo Harada, Seitaro Nomura, Norihiko Takeda, Brian N. Finck, Haruhiro Toko, Issei Komuro

**Affiliations:** 1 Department of Anesthesiology, The First Affiliated Hospital, Zhejiang University School of Medicine, Hangzhou, China.; 2 Department of Cardiovascular Medicine, Graduate School of Medicine, and; 3 Department of Frontier Cardiovascular Science, Graduate School of Medicine, The University of Tokyo, Tokyo, Japan.; 4 Cardiovascular Biobank Research Center, International University of Health and Welfare, Tokyo, Japan.; 5 Department of Advanced Clinical Science and Therapeutics, Graduate School of Medicine, The University of Tokyo, Tokyo, Japan.; 6 Center for Human Nutrition, Department of Medicine, Washington University School of Medicine, St. Louis, Missouri, USA.

**Keywords:** Cardiology, Metabolism, Cardiovascular disease

## Abstract

Impaired cardiac lipid metabolism has been reported to cause heart failure. Lipin1, a multifunctional protein, is a phosphatidate phosphatase that generates diacylglycerol from phosphatidic acid and a transcriptional cofactor that regulates lipid metabolism-related gene expression. Here, we investigated the roles of lipin1 in cardiac remodeling after myocardial infarction (MI). The expression levels of lipin1 significantly decreased in cardiomyocytes of the human failing heart and murine ischemic myocardium. Cardiomyocyte-specific *Lpin1* knockout (cKO) mice showed left ventricle enlargement and reduced fractional shortening after MI, compared with control mice. This was accompanied by elevated cardiac fibrosis, accumulation of reactive oxygen species, and increased expression of inflammatory cytokines. In contrast, cardiomyocyte-specific *Lpin1* overexpression (cOE) mice showed reduced fibrosis and inflammation and improved cardiac function compared with control mice. Cardiac lipid droplets (LDs) were reduced after MI in WT mouse hearts and were further downregulated in the hearts of cKO mice with a decrease in triacylglycerol and free fatty acid content, while cOE mice hearts exhibited increased LDs and lipid content. Expression levels of genes involved in fatty acid oxidation, such as *Ppargc1a* (PGC1A) and *Acaa2*, were decreased and increased in the MI hearts of cKO mice and cOE mice, respectively. These results suggest the protective role of lipin1 against ischemic injury by maintaining lipid metabolism in ischemic cardiomyocytes.

## Abstract 2

Myocardial lipin1 protects the heart from ischemic injury by regulating lipid metabolism.

## Graphical abstract

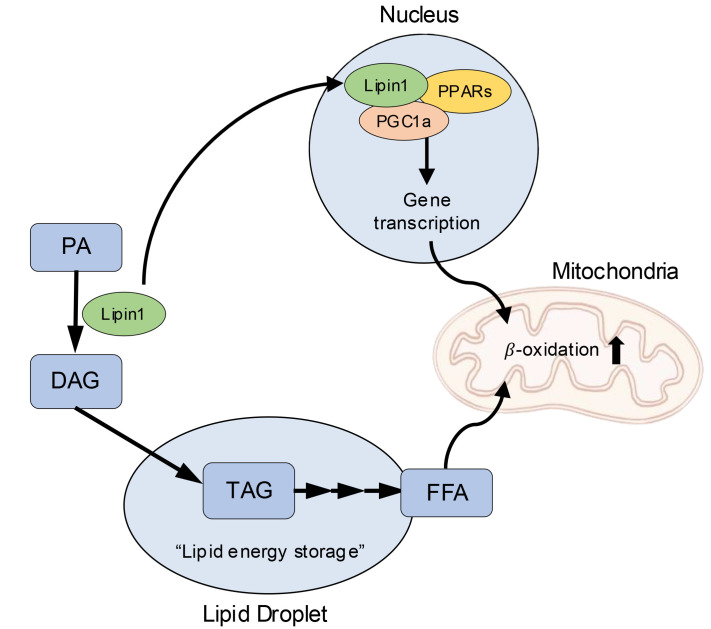

## Introduction

Medical advancements have improved survival rates in patients with acute myocardial infarction (MI), but the incidence of heart failure after MI has increased (1–4). Left ventricular (LV) remodeling, characterized by ventricular dilatation and increased interstitial fibrosis, is pivotal to the progression of heart failure (5, 6). Despite effective pharmacological therapies, heart failure remains a leading global cause of death (7–9). Thus, investigating innovative approaches to prevent LV remodeling after MI is crucial.

The heart relies on diverse substrates for its high metabolic demands. In a physiological state, myocardial fatty acid oxidation provides approximately 70% of energy requirements. However, in pathological conditions such as cardiac hypertrophy and heart failure, fatty acid oxidation is reduced, leading to a compensatory shift to glycolysis (10, 11). Given that glycolysis is insufficient to supply the ATP required for normal heart contraction, disruption of fatty acid oxidation homeostasis is closely associated with the development of heart failure (12–14).

A mutation of the gene *Lpin1*, which encodes lipin1, was identified in a mouse line showing dystrophic phenotypes, including deficiencies in triacylglycerol (TAG) production (15, 16). Lipin1 is recognized as a multifaceted molecule governing at least 2 distinct lipid metabolism pathways. In the cytoplasm, lipin1 acts as a phosphatidic acid phosphohydrolase (PAP) enzyme, catalyzing the conversion of phosphatidic acid (PA) into diacylglycerol (DAG), an essential precursor for TAG, phosphatidylethanolamine, and phosphatidylcholine synthesis (17). TAG, a primary energy store with a crucial role in sustaining energy equilibrium, resides mainly within lipid droplets (LDs), which consist of a TAG core with a surface monolayer of phospholipids and associated LD proteins (18, 19). Additionally, lipin1 exhibits transcriptional coregulatory activity, forming a complex with peroxisome proliferator–activated receptor–γ coactivator-1α (PGC1A) and peroxisome proliferator–activated receptor α or γ (PPARA/G) (20). The lipin1-PGC1A-PPARA complex regulates the expression of genes linked to mitochondrial fatty acid oxidation, such as carnitine palmitoyltransferase 1b (*Cpt1b*) and acetyl-coenzyme A acyltransferase 2 (*Acaa2*), which are essential for normal cardiac function (21–24).

We and other researchers have observed a decrease in lipin1 expression in both murine and human failing hearts (25–27). In the present study, single-nucleus RNA-Seq analysis reveals distinctive expression of *LPIN1* in cardiomyocytes within human cardiac tissue, along with significant downregulation of its expression in heart failure. Consequently, we investigated the functional role of lipin1 in LV remodeling after MI using 2 distinct cardiac-specific *Lpin1* transgenic mouse models: cardiomyocyte-specific *Lpin1* knockout (αMHC-Cre^+/−^ *Lpin1*^fl/fl^; cKO) and overexpression (αMHC-Cre^+/−^ *Lpin1*^STOP/STOP^; cOE) mice. We evaluated cardiac function and remodeling after MI in these mice, as well as LD distribution, lipid content, and metabolism in the heart.

## Results

### A downregulation of myocardial LPIN1 in human heart failure.

**Figure 1:**
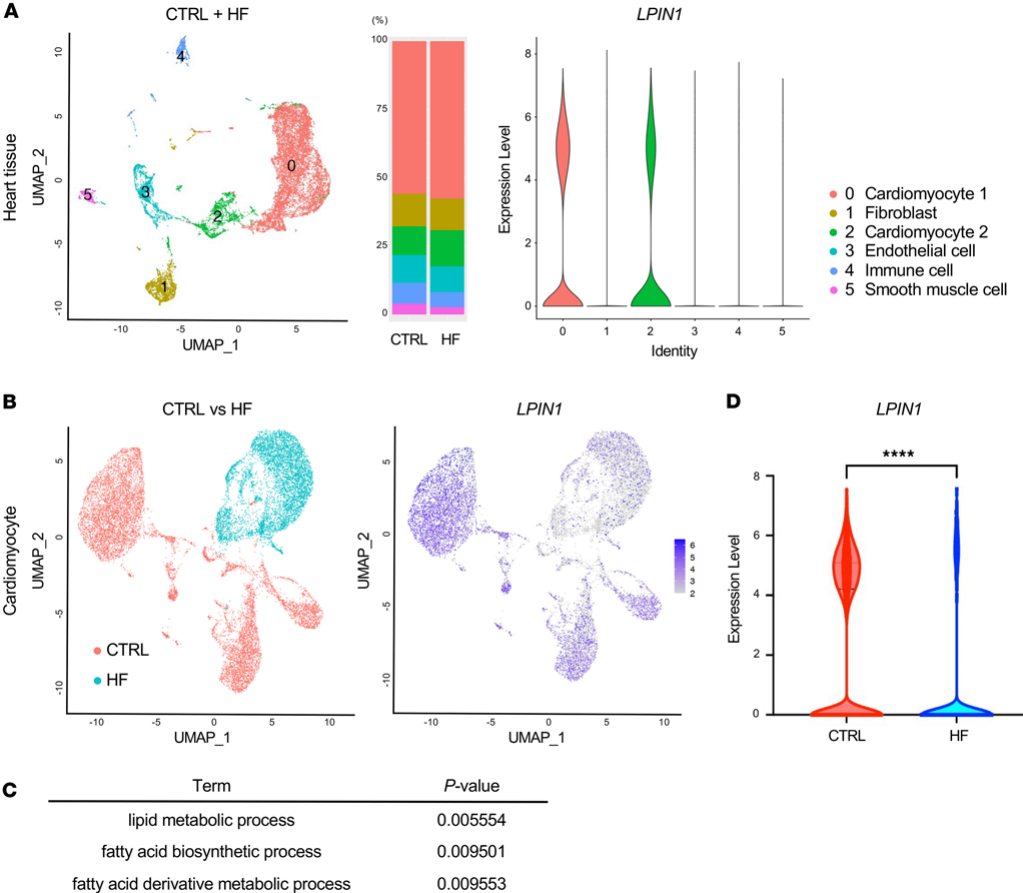
Single-cell analysis identifies cardiac *LPIN1*. (**A**) Uniform Manifold Approximation and Projection (UMAP) plot of single-cell transcriptomes of cardiomyocytes and noncardiomyocytes from 3 patients with heart failure (HF) and 3 control (CTRL) patients. The heart tissue cells were divided into 6 clusters which are color-coded by cell type. Cardiac *LPIN1* is mainly expressed in cardiomyocytes. (**B**) Feature plots of expression distribution for cardiomyocytes (clusters 0 and 2). The expression level of *LPIN1* is color-coded. (**C**) Pathway analysis indicated significant changes in gene expression related to lipid and fatty acid metabolism pathways between HF and CTRL cardiomyocytes. (**D**) The expression level of cardiac *LPIN1* was decreased in patients with HF compared with the CTRL.

Single-nucleus RNA-Seq analysis was conducted on cardiac biopsy samples obtained from 3 patients with heart failure and 3 control patients. Approximately 50%–60% of each tissue sample consisted of cardiomyocytes expressing characteristic genes such as *TTN* (Figure 1A and Supplemental Figure 1, A and B; supplemental material available online with this article; https://doi.org/10.1172/jci.insight.183334DS1). Subcluster analysis using cardiomyocytes revealed substantial disparities in gene expression characteristics between the control and heart failure groups (Figure 1B). Pathway analysis indicated significant downregulation of gene expression related to lipid metabolism pathways in cardiomyocytes of patients with heart failure (Figure 1C). *LPIN1*, identified as a representative differentially expressed gene (DEG) in these pathways, exhibited selective expression in cardiomyocytes (Figure 1A and Supplemental Figure 2), with a marked decrease observed in cardiomyocytes from patients with heart failure (Figure 1, B and D). This downregulation was similarly observed in the ischemic cardiomyopathy case among the 3 patients with heart failure (Supplemental Figure 3A). In parallel, genes associated with PPARG signaling were also downregulated in cardiomyocytes from failing hearts (Supplemental Figure 3B). These findings suggest that lipin1 is specifically expressed in cardiomyocytes within the heart and that its expression is markedly reduced in heart failure.

### The role of lipin1 in cardiac remodeling after MI.

**Figure 2:**
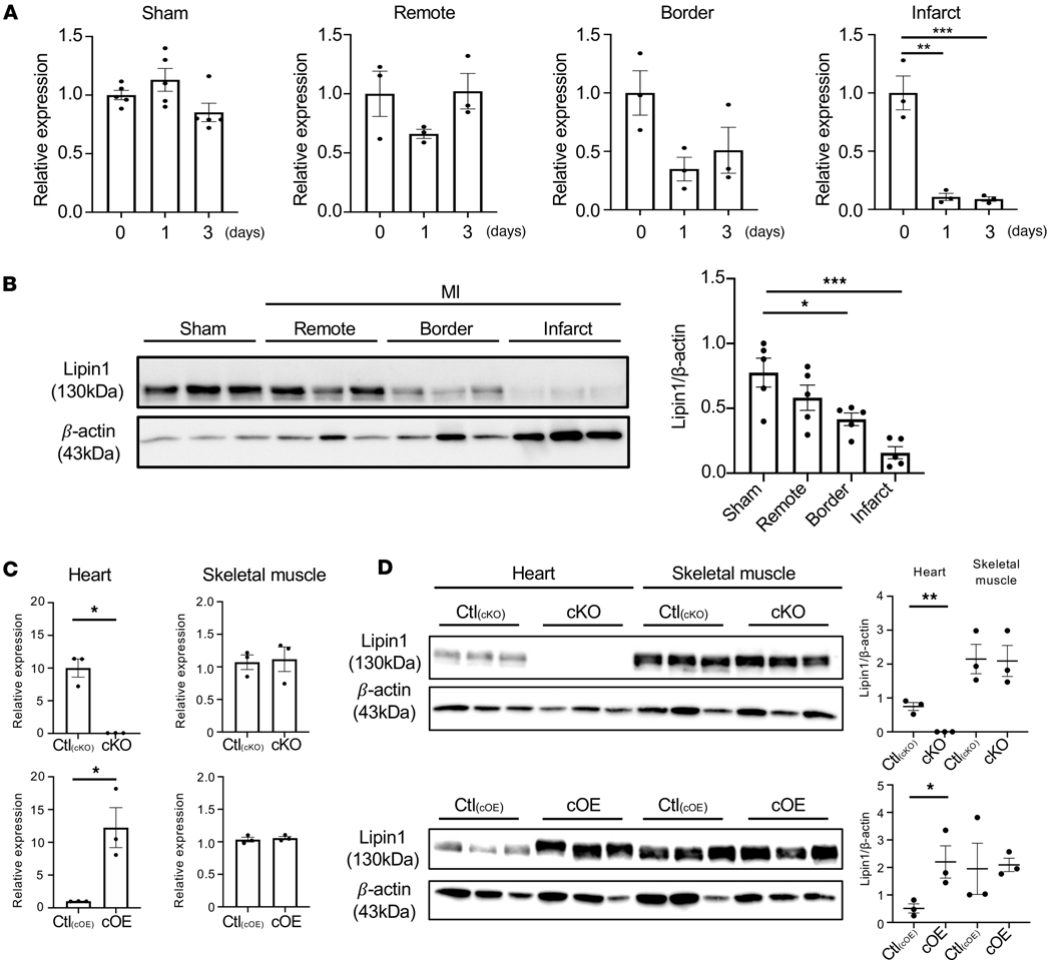
*Lpin1*/lipin1 expression levels after MI surgery. (**A**) *Lpin1* mRNA expression was measured by quantitative PCR and exhibited no significant difference in sham group, but it showed diminished levels 1 day and 3 days after MI surgery in the border and remote zones of WT mice compared with pre-MI levels. *n* = 5 each (Sham), *n* = 3 each (MI). Data are presented as the mean ± SEM. **P < 0.01, ***P < 0.001 by ordinary 1-way ANOVA. (**B**) Lipin1 protein expression, assessed through Western blot 1 week after MI, showed reduced abundance in the border and remote zones. *n* = 5 each. Data are presented as the mean ± SEM. *P < 0.05, ***P < 0.001 by ordinary 1-way ANOVA. (**C**) The expression level of *Lpin1* was measured in both heart and skeletal muscle of *Lpin1* transgenic mice. *Lpin1* mRNA expression was only changed in hearts, but there were no significant difference in skeletal muscle. *n* = 3 each. Data are presented as the mean ± SEM. *P < 0.05 by unpaired 2-tailed *t* test. (**D**) Lipin1 protein expression, assessed through Western blot using heart or skeletal muscle of *Lpin1* transgenic mice, displayed no significant difference in skeletal muscle but increased or decreased significantly in the cKO/cOE heart. *n* = 3 each. Data are presented as the mean ± SEM. *P < 0.05, **P < 0.01 by unpaired 2-tailed *t* test.

Consequently, we investigated the role of myocardial lipin1 during the development of ischemic heart failure using mouse MI models. Lpin1 mRNA expression levels showed no significant time-dependent changes in sham-operated hearts, whereas they were markedly decreased in ischemic WT hearts after MI, particularly in the infarct zone (Figure 2A). Correspondingly, lipin1 protein expression was reduced in both the border and infarct zones 1 week after MI (Figure 2B). Using the Cre/lox P system, cKO and cOE mice were generated. cKO mice exhibited a significant reduction of lipin1 expression at both mRNA and protein levels in the hearts but not in skeletal muscle (Figure 2, C and D). Conversely, expressions of lipin1 mRNA and protein significantly increased in cOE mice hearts compared with Cre^−^ controls, with no discernible change in skeletal muscle (Figure 2, C and D).

**Figure 3:**
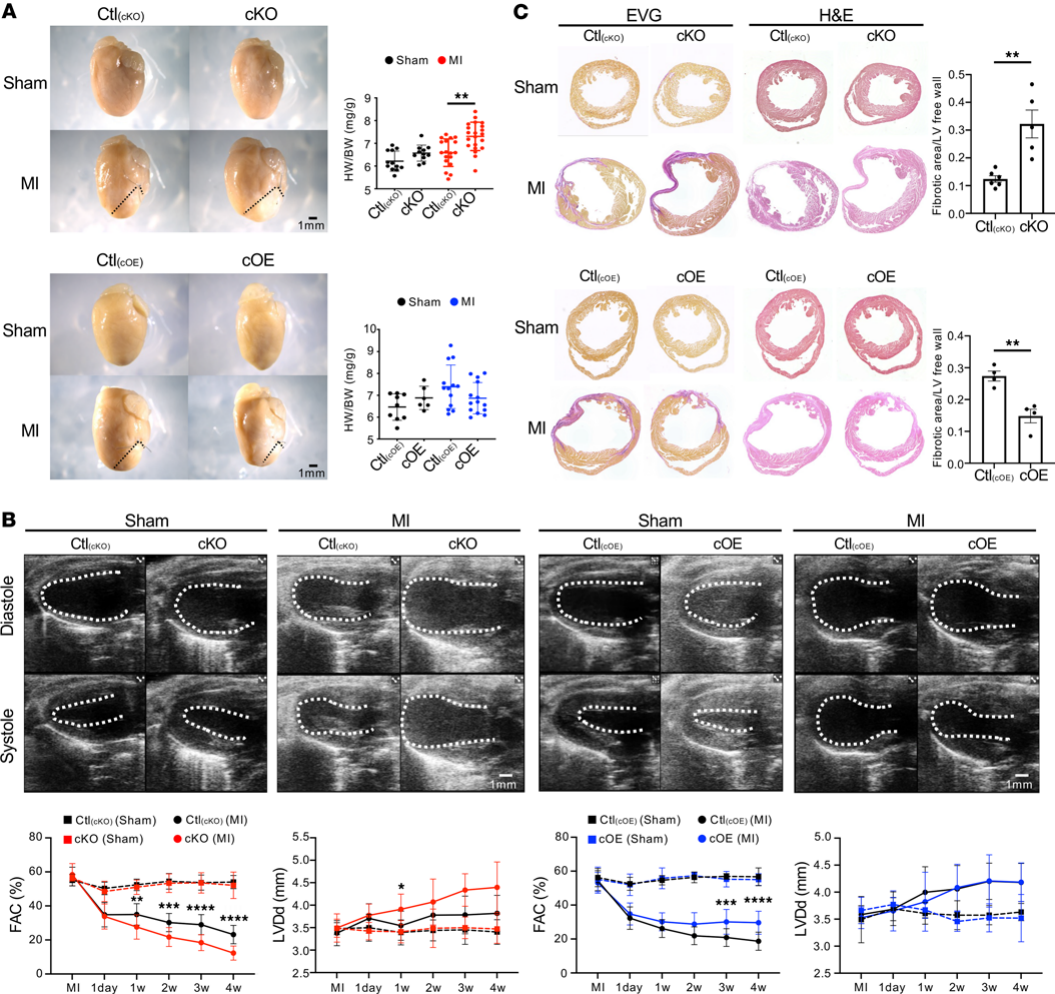
Cardiac function dynamics after MI surgery. (**A**) Four weeks after MI, cKO mice showed exacerbated remodeling and a significantly elevated HW/BW ratio compared with their littermates. *n* = 12 [Ctl_(cKO)_, sham], *n* = 11 (cKO, sham), *n* = 18 [Ctl_(cKO)_, MI], *n* = 21 (cKO, MI). Data are presented as the mean ± SEM. **P < 0.01 by ordinary 1-way ANOVA. cOE mice displayed milder remodeling relative to their littermate controls, maintaining a comparable heart weight/body weight (HW/BW) ratio. *n* = 9 [Ctl_(cOE)_, sham], *n* = 6 (cOE, sham), *n* = 13 [Ctl_(cOE)_, MI], *n* = 14 (cOE, MI). Data are presented as the mean ± SEM by ordinary 1-way ANOVA. Scale bar: 1mm. (**B**) B-mode echocardiography images at diastole and systole over 4 weeks after MI displayed endocardial surfaces, revealing left ventricular end-diastolic diameter (LVDd) and fractional area change (FAC) in cKO/cOE and their littermate controls. cKO mice showed a decreased FAC with increased LVDd after MI than their littermates. *n* = 12 [Ctl_(cKO)_, sham], *n* = 11 (cKO, sham), *n* = 16–18 [Ctl_(cKO)_, MI], *n* = 20-21 (cKO, MI). Data are presented as the mean ± SEM. *P < 0.05, **P < 0.01, ***P < 0.001, ****P < 0.0001 by 2-way ANOVA. cOE mice showed an increased FAC after MI than their littermates. *n* = 9 [Ctl_(cOE)_, sham], *n* = 6 (cOE, sham), *n* = 13 [Ctl_(cOE)_, MI], *n* = 14 (cOE, MI). Scale bar: 1mm. (**C**) H&E and EVG staining of 4-week infarct hearts showcased bigger fibrosis size (fibrotic area/left ventricular free wall) in cKO mice compared with their littermates, underscoring the severity of fibrosis. *n* = 6 [Ctl_(cKO)_], *n* = 5 (cKO). Data are presented as the mean ± SEM. **P < 0.01 by unpaired 2-tailed *t* test. In contrast, cOE mice exhibited diminished fibrosis size compared with their littermates. *n* = 4 [Ctl_(cOE)_], *n* = 4 (cOE). Data are presented as the mean ± SEM. **P < 0.01 by unpaired 2-tailed *t* test.

Four weeks after MI surgery, the heart weight/body weight (HW/BW) ratio of cKO mice exceeded that of their control littermates [αMHC-Cre^−/−^ *Lpin1*^fl/fl^; Ctl_(cKO)_], while no difference was observed between cOE mice and their control littermates [αMHC-Cre^−/−^ *Lpin1*^STOP/STOP^; Ctl_(cOE)_] (Figure 3A). Echocardiography showed no difference among sham-operated cKO mice, cOE mice, and their control littermates in terms of LV end-diastolic diameter (LVDd) and LV fractional area change (LVFAC) (Figure 3B). After MI, however, cKO mice displayed a significantly enlarged LVDd and decreased LVFAC compared with Ctl_(cKO)_ mice (Figure 3B). Conversely, LVFAC was higher in cOE mice than in Ctl_(cOE)_ mice (Figure 3B). Elastica van Gieson (EVG) staining revealed extended fibrotic areas in the infarcted heart of cKO mice compared with Ctl_(cKO)_ mice, whereas cOE mice showed diminished fibrotic regions (Figure 3C), suggesting that lipin1 inhibits remodeling and maintains cardiac function after MI.

### Lipin1 maintains lipid utilization within the myocardium after MI.

**Figure 4:**
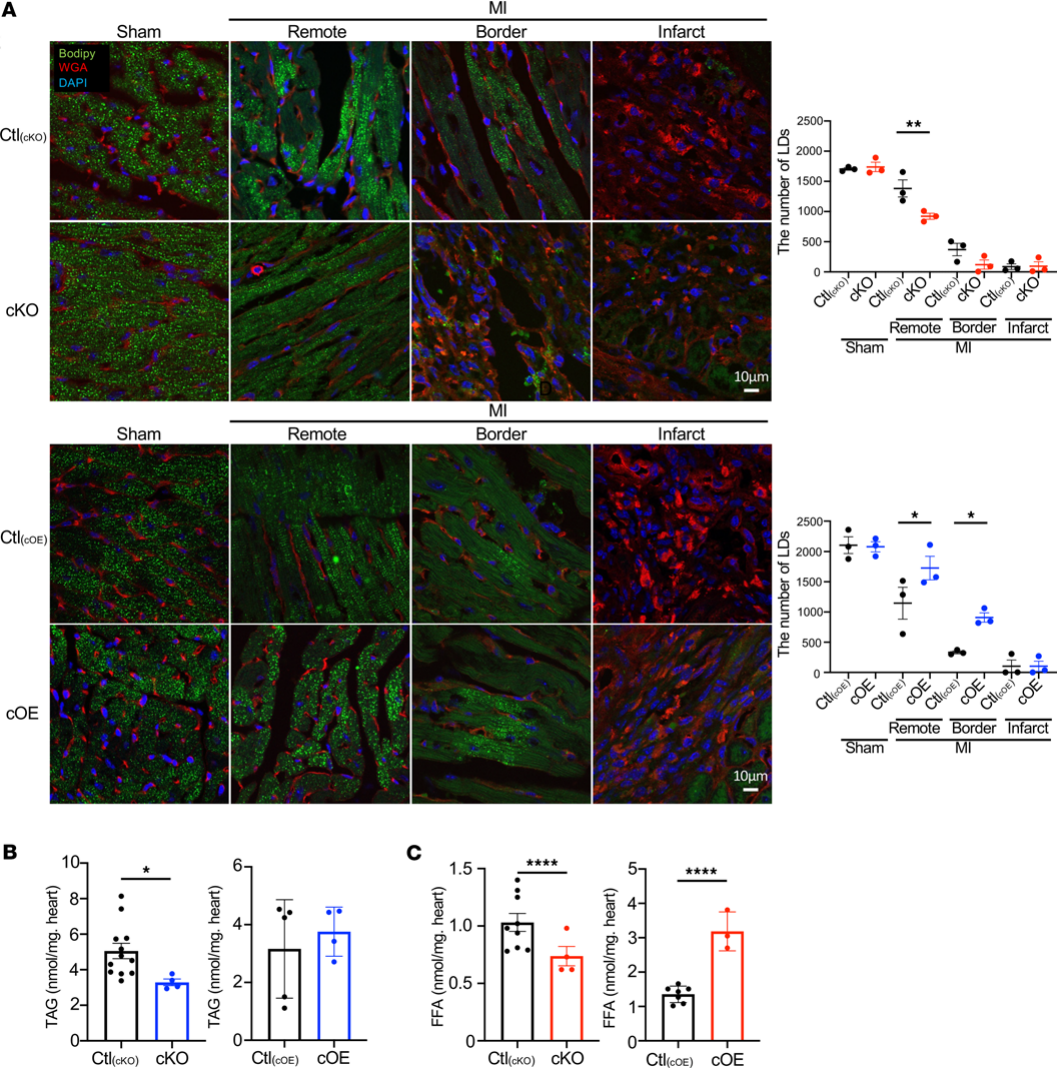
Accumulation of lipid droplets in infarcted hearts. (**A**) One week after MI, hearts were Bodipy-stained to calculate lipid droplet numbers using ImageJ. LDs were stained by Bodipy in green, the nucleus was stained by DAPI in blue, and membranes were stained by WGA in red. While no disparities were noted in the sham group, cKO mice displayed fewer lipid droplets in the remote zone compared with control mice, whereas cOE mice exhibited more lipid droplets in the border and remote zones. *n* = 3 each. Data are presented as the mean ± SEM. *P < 0.05, **P < 0.01 by ordinary 1-way ANOVA. Scale bars: 10 μm. (**B**) The content in infarct hearts of TAG was observed, which showed that the storage of TAG was reduced after MI in cKO mice compared with their littermate controls. On the contrary, there is an increased tendency of TAG in cOE mice than the controls. *n* = 12 [Ctl_(cKO)_], *n* = 4 (cKO), *n* = 5 [Ctl_(cOE)_], *n* = 4 (cOE). Data are presented as the mean ± SEM. *P < 0.05 by unpaired 2-tailed *t* test. (**C**) Reduced FFA storage was observed in cKO mice. Conversely, cOE mice exhibited increased FFA expression after MI. *n* = 9 [Ctl_(cKO)_], *n* = 4 (cKO), *n* = 7 [Ctl_(cOE)_], *n* = 3 (cOE). Data are presented as the mean ± SEM. ****P < 0.0001 by unpaired 2-tailed *t* test.

Lipin1 produces DAG as a PAP enzyme and regulates expression of lipid metabolism–related genes as a transcriptional cofactor. Therefore, we next examined the importance of lipid metabolism processes in the lipin1-mediated protective mechanism against cardiac remodeling after MI. LDs, recognized as pivotal reservoirs for TAG, play a critical role in cardiac function preservation (28). Although no significant differences were observed among cKO mice, cOE mice, and their control littermates in sham-operated groups, cKO mice hearts showed a significant reduction in LD abundance labeled by Bodipy staining after MI, particularly in the remote zone. Conversely, cOE mice exhibited an elevation in LD numbers within the remote and border zones (Figure 4A). TAG content was decreased in cKO mice, and the levels of its downstream catabolic product, free fatty acids (FFAs), were decreased in cKO mice and increased in cOE mice (Figure 4, B and C).

**Figure 5:**
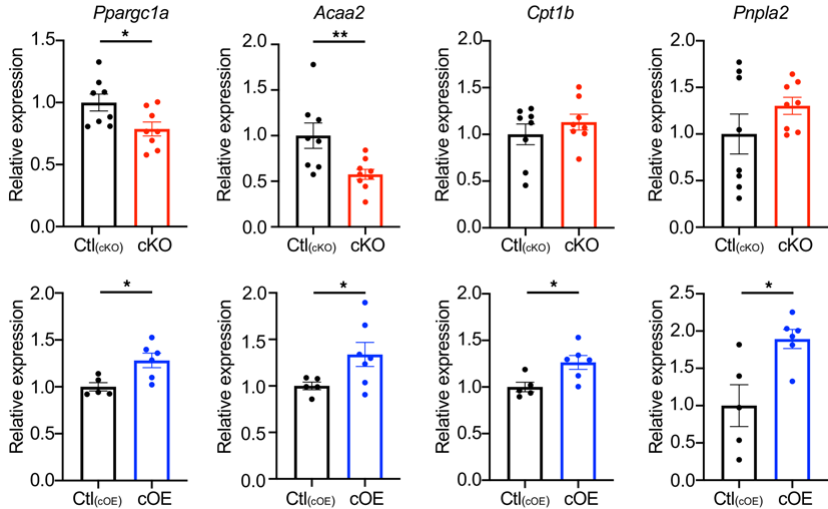
Expression levels of fatty acid oxidation–related genes. Expression levels of *Ppargc1a*, *Acaa2*, *Cpt1b*, and *Pnpla2* were evaluated both in cKO and cOE hearts. The expression of *Ppargc1a* and *Acaa2* was diminished in cKO mice hearts, whereas the expression of *Ppargc1a*, *Acaa2*, *Cpt1b*, and *Pnpla2* was elevated in cOE mice hearts. *n* = 8 [Ctl_(cKO)_], *n* = 8-9 (cKO), *n* = 5 each [Ctl_(cOE)_], *n* = 6–7 (cOE). Data are presented as the mean ± SEM. *P < 0.05, **P < 0.01 by unpaired 2-tailed *t* test.

Additionally, expression levels of genes involved in fatty acid oxidation, such as *Ppargc1a* (PGC1A) and *Acaa2*, decreased in MI hearts of cKO mice (Figure 5), while cOE mice hearts showed increased expression of *Ppargc1a*, *Acaa2*, *Cpt1b*, and *Pnpla2* (ATGL) (Figure 5). These results suggest that lipin1 facilitates enhanced TAG production and storage within LDs and further exerts regulatory influence over the lipid oxidation pathway.

### Lipin1 attenuates inflammation and oxidative stress in the MI hearts.

**Figure 6:**
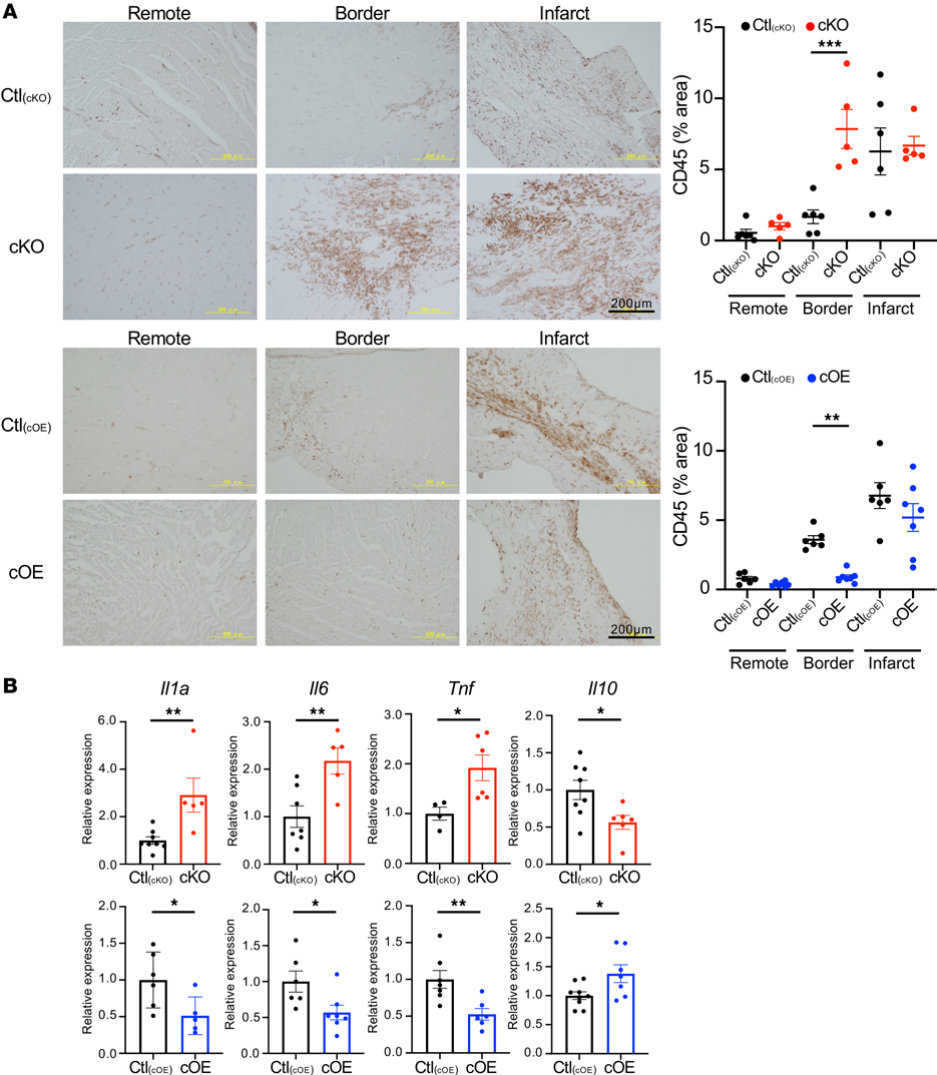
Inflammation after MI surgery. (**A**) One week after MI, hearts were CD45 stained, and ImageJ was employed to calculate positive cell numbers. The border zone of cKO mice exhibited more positive cells than the control mice, while cOE mice displayed fewer. *n* = 6 each [Ctl_(cKO)_], *n* = 5 each (cKO), *n* = 6 each [Ctl_(cOE)_], *n* = 7 each (cOE). Data are presented as the mean ± SEM. **P < 0.01, ***P < 0.001 by ordinary 1-way ANOVA. Scale bars: 200 μm. (**B**) One week after MI, proinflammatory and antiinflammatory factor expression levels were measured. In cKO hearts, the expression of proinflammatory factors including *Il1a*, *Il6*, and *Tnf* was increased, whereas the expression of antiinflammatory factor *Il10* was decreased. In contrast, the expression of proinflammatory factors was decreased, whereas the expression of antiinflammatory factor was increased in cOE hearts. *n* = 4–8 [Ctl_(cKO)_], *n* = 5–6 (cKO), *n* = 6–9 [Ctl_(cOE)_], *n* = 5–7 (cOE). Data are presented as the mean ± SEM. *P < 0.05, **P < 0.01 by unpaired 2-tailed *t* test.

Dysregulation of cardiac lipid metabolism has been reported to trigger cardiac inflammation (29, 30). In the border zone, cKO mice exhibited increased CD45^+^ leukocyte accumulation, whereas a decrease in leukocytes was observed in cOE mice (Figure 6A). Gene expression levels of inflammatory cytokines, including *Il1a*, *Il6*, and *Tnf*, in the infarcted hearts were upregulated in cKO mice and downregulated in cOE mice compared with each control (Figure 6B). Conversely, *Il10* expression was downregulated in cKO mice and upregulated in cOE mice (Figure 6B).

**Figure 7:**
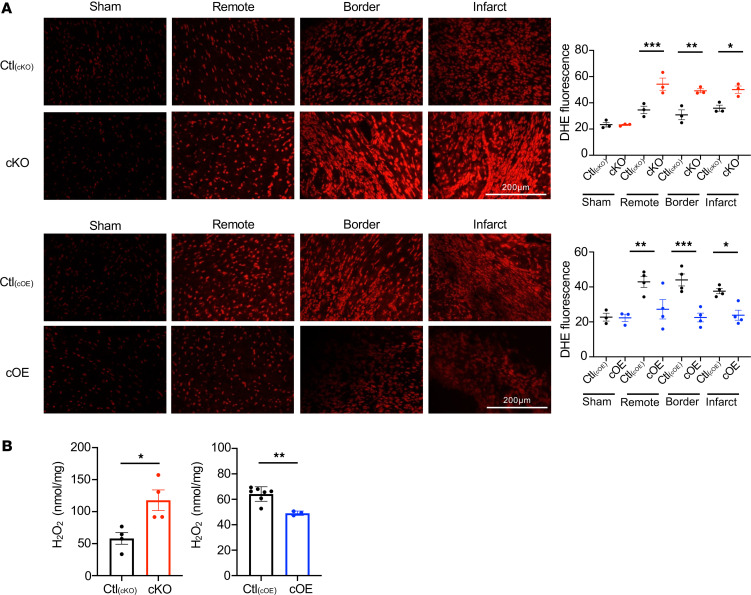
Oxidative stress after MI surgery. (**A**) Examination of DHE fluorescence in different zones 1 week after MI or sham surgery showed no significant difference in sham group. In contrast, cKO hearts showed increased DHE fluorescence following MI, whereas cOE hearts showed a significant decrease compared with controls. *n* = 3 each [Ctl_(cKO)_], *n* = 3 each (cKO), *n* = 3 [Ctl_(cOE)_, Sham], *n* = 3 (cOE, Sham), *n* = 4 each [Ctl_(cOE)_, MI], *n* = 4 each (cOE, MI). Data are presented as the mean ± SEM. *P < 0.05, **P < 0.01, ***P < 0.001 by ordinary 1-way ANOVA. Scale bars: 200 μm. (**B**) H_2_O_2_ levels were increased in cKO hearts, while decreased in cOE hearts. *n* = 4 [Ctl_(cKO)_)] *n* = 4 (cKO); *n* = 7 [Ctl_(cOE)_], *n* = 3 (cOE). Data are presented as the mean ± SEM. *P < 0.05, **P < 0.01 by unpaired 2-tailed *t* test.

To examine lipin1’s effect on oxidative stress in MI hearts, dihydroethidium (DHE) staining and hydrogen peroxide (H_2_O_2_) content were assessed. DHE fluorescence showed no significant differences among genotypes in sham-operated groups. In contrast, in the remote, border, and infarct zones, cKO mice exhibited increased DHE fluorescence, whereas cOE mice showed reduced fluorescence compared with their controls (Figure 7A). Concurrently, H_2_O_2_ levels were increased and decreased in cKO and cOE mice, respectively (Figure 7B). These findings underscore lipin1’s efficacy in limiting inflammation and oxidative stress in MI hearts.

### Oxidative stress and cardiac dysfunction in lipin1 deficiency.

**Figure 8:**
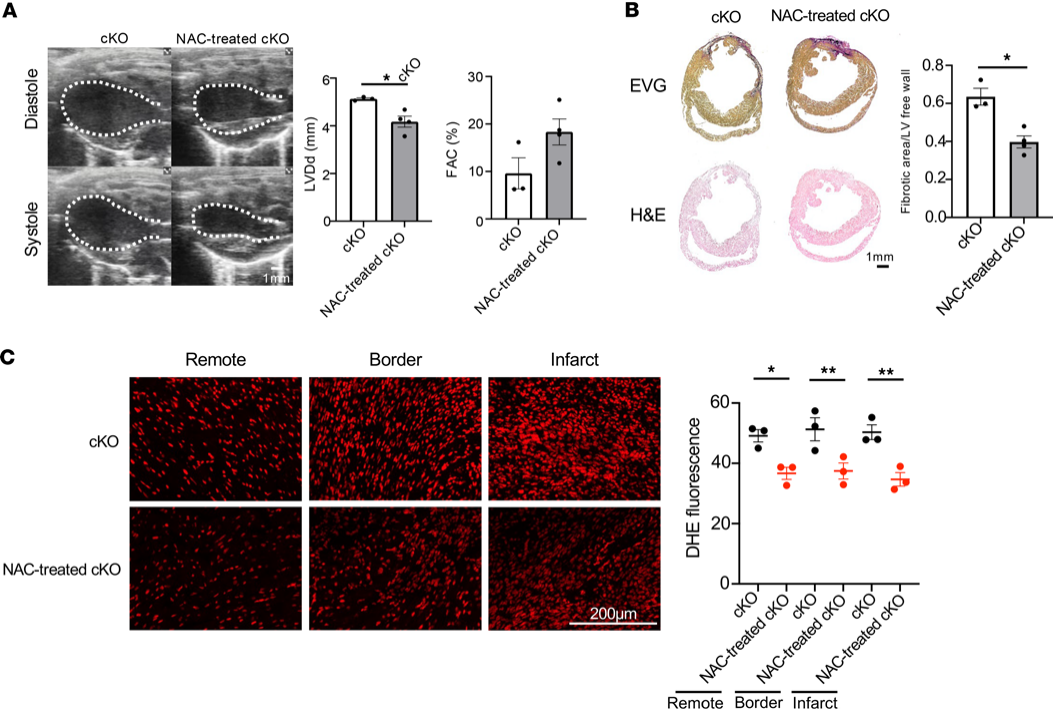
NAC treatment in cKO mice after MI. (**A**) Echocardiographic analysis of cardiac function at 4 weeks after MI. NAC-treated cKO mice showed significantly improved systolic function, with decreased LVDd and increased FAC compared with untreated cKO mice. *n* = 3 (cKO), *n* = 4 (NAC-treated cKO). Data are presented as the mean ± SEM. *P < 0.05 by unpaired 2-tailed *t* test.Scale bar: 1mm. (**B**) EVG and H&E staining of heart sections at 4 weeks after MI. NAC treatment reduced fibrotic area in cKO hearts. Fibrotic area to LV free wall was measured. *n* = 3 (cKO), *n* = 4 (NAC-treated cKO). Data are presented as the mean ± SEM. *P < 0.05 by unpaired 2-tailed *t* test. (**C**) DHE staining showed reduced ROS levels in NAC-treated cKO mice at infarct, border, and remote zones. *n* = 3 (cKO), *n* = 3 (NAC-treated cKO). Data are presented as the mean ± SEM. *P < 0.05, **P < 0.01 by ordinary 1-way ANOVA. Scale bar: 200 μm.

To further investigate the involvement of oxidative stress in the impaired cardiac function observed in cKO mice after MI, we treated cKO mice with N-acetylcysteine (NAC), an antioxidant agent, and assessed cardiac structure and function 4 weeks after MI. Echocardiographic analysis revealed that NAC treatment significantly reduced LV enlargement compared with untreated cKO mice, and fractional area change (FAC) showed a trend toward improvement (Figure 8A). Histological analysis using EVG and H&E staining demonstrated a significant reduction in myocardial fibrosis in NAC-treated cKO hearts relative to untreated controls (Figure 8B). DHE staining revealed markedly decreased fluorescence in the infarct, border, and remote zones of NAC-treated cKO hearts, indicating reduced reactive oxygen species (ROS) levels (Figure 8C). These results suggest that antioxidant treatment mitigates pathological cardiac remodeling after MI associated with cardiomyocyte-specific lipin1 deficiency.

## Discussion

Under ischemic conditions, the heart undergoes a metabolic shift from lipid to glucose as its primary energy source, leading to a decrease in lipid oxidation and a compensatory transition toward glycolysis (10, 11). Disruption of lipid oxidation homeostasis is closely associated with the development of heart failure (12–14). Single-nucleus RNA-Seq analysis revealed reduced expression of lipin1, a multifaceted molecule governing lipid metabolism pathway, in cardiomyocytes of the human failing heart. This study highlights the regulatory role of lipin1 in lipid metabolism of ischemic myocardium.

Using 2 distinct cardiac-specific *Lpin1* transgenic mouse models, knockout and overexpression mice, we demonstrated that lipin1 preserves cardiac function after MI by regulating lipid metabolism and sustaining a sufficient energy supply through lipid oxygenation, meeting myocardial demand. Lipin1 increased LD accumulation in the myocardium, likely through its enzymatic action promoting DAG generation from PA. Lipin1 also increases the expression levels of lipid metabolism–related molecules, including *Ppargc1a*, *Pnpla2*, and *Cpt1b*, contributing to mitochondrial biogenesis, and β-oxidation. There is a study on the liver reporting that lipin1 regulates fatty acid oxidation in the cytoplasmic and gene expression in the nucleus (20), similar to our observations in the myocardium.

Our data suggest that lipin1 is essential for maintaining lipid availability to meet the metabolic demands of the ischemic heart. This is achieved by facilitating TAG and LD formation, which in turn supplies FFAs as an energy source for mitochondria. Infarcted hearts of cKO mice exhibited reduced levels of TAG and LD. In contrast, Chambers et al. reported increased TAG content in the hearts of cKO mice under basal conditions (26). This discrepancy may be attributed to differences in experimental context: their study examined hearts under physiological conditions, whereas ours focused on post-MI hearts, a distinctly pathological state. It is plausible that these divergent cardiac contexts underlie the contrasting findings. Notably, *Lpin1* overexpression did not affect cardiac function under nonischemic conditions, indicating that lipin1 supports lipid utilization through LD formation without inducing lipotoxicity (31, 32). Our study also revealed that lipin1 confers substantial benefits to the ischemic heart by attenuating inflammation and oxidative stress. In cooperation with PGC1A, lipin1 may help improve mitochondrial function, thereby contributing to the reduction of excessive ROS production and inflammation following MI (31–34). However, further studies are needed to elucidate the specific effects of lipin1 overexpression and deficiency on mitochondrial function.

In conclusion, our study provides detailed mechanistic insights into cardiac responses to ischemic stress, highlighting the crucial role of lipin1–mediated lipid metabolism in the ischemic heart. Our findings suggest that lipin1 holds potential as a therapeutic target against MI, the persistent high morbidity of cardiovascular diseases despite advancements in interventions and pharmacological treatments.

## Methods

### Sex as a biological variable.

Human heart tissues were collected from male and female patients. Both male and female mice were used in this study.

### Single-nucleus RNA-Seq of human heart.

Cardiac biopsy samples from patients with heart failure undergoing LV assist device implantation were immediately fresh-frozen. Heart tissue samples for the control group were obtained from patients who died due to noncardiogenic reasons and subsequently underwent autopsy. Patient information is shown in Supplemental Table 1. We isolated nuclei with the Singulator 100 System (#100-067-764, S2 Genomics) using Singulator Nuclei Isolation Kit (#100-060-817, S2 Genomics). Then, 5,000 nuclei were prepared to a concentration of 1,000 nuclei/μL and loaded into the Chromium Controller (10x Genomics), and single-nucleus cDNA libraries were generated using a Chromium 3′ v3 Chemistry Kit (#PN-1000075, 10x Genomics). Libraries were sequenced on a NovaSeq 6000 System (Illumina) using a NovaSeq S4 Reagent Kit (200 cycles; #20027466, Illumina).

### Single-nucleus RNA-Seq analysis of human heart.

Raw FASTQ files were processed for each sample with Cell Ranger software (ver 7.1.0, 10x Genomics) against the Cell Ranger GRCh38 human reference genome. Raw mapped counts were used as the input for data processing with the Seurat R package (version 4.1.2) (35). To exclude ambient RNA contamination, the data were processed using CellBender (36). We removed cells with detected genes less than 400, detected counts less than 5000, more than 20,000, and with mitochondrial gene content greater than 15%. Following the filtering step, we normalized read counts using the ‘NormalizeData’ function (10,000 default scale factor) separately for each dataset. We used the ‘FindVariableFeatures’ function to identify highly variable features for downstream analysis and integrated them using the ‘FindIntegrationAnchors’ and ‘IntegrateData’ functions. The integrated data were used for dimensionality reduction and cluster detection. We performed linear regression using the ‘ScaleData’ function and linear dimensionality reduction using the ‘RunPCA’ function. Twenty principal components were used for downstream graph-based supervised clustering into distinct populations using the ‘FindClusters’ function and uniform manifold approximation and projection (UMAP) dimensionality reduction was performed to project the cell population onto 2 dimensions using the ‘RunUMAP’ function. DEGs were detected by using the ‘FindMarkers’ function (log2fc.threshold > 0.25 and p_val_adj < 0.05). Using the obtained marker data, a heat map was drawn using the ‘DoHeatmap’ function. After we retrieved the data for each cell type, we used the ‘FindVariableFeatures’ function to identify highly variable features for downstream analysis. We then used the ‘ScaleData’, ‘RunPCA’, ‘FindClusters’, ‘RunUMAP’, and ‘FindMarkers’ functions on the subsetted every cell cluster. The DEGs were identified according to the adjusted *P* values (p_val_adj < 0.05) and then subjected to pathway analysis using Metascape (37).

### Generation of transgenic mice.

The selected mouse strains shared a common genetic foundation with the C57BL/6 lineage. WT mice were procured from the CLEA Japan (Tokyo, Japan). The Cre/loxP system was employed to generate conditional *Lpin1* knockout and *Lpin1* overexpression mice. The generation of *Lpin1*^fl/fl^ mice has previously been described ref. 38 (B6[Cg]-Lpin1^tm1c[EUCOMM]Hmgu/FincJ^; The Jackson Laboratory, stock no. 032117). The generation of *Lpin1*^STOP/STOP^ mice has also previously been described (39). Briefly, *Lpin1*^STOP/STOP^ mice were generated by knocking in a cassette containing a mouse lipin1 cDNA into the ROSA26 locus by using TALENS. This cassette contains, in sequence, the constitutive chicken α-actin promoter, 3 stop codons flanked by LoxP sites, and the cDNA for mouse lipin1. ES cells with this allele knocked in were injected into developing mouse embryos. Crossing *Lpin1*^STOP/STOP^ mice with mice expressing Cre recombinase removes the stop codons from the cassette and allows for tissue-specific overexpression of lipin1. αMHC-Cre mice (stock no.: #009074, stock name: Tg [Myh6-cre]1Jmk/J) were obtained from The Jackson Laboratory (Bar Harbor, ME, USA). For the entirety of our experimental procedures, non-Cre-expressing littermates were deployed as a representative control group.

### Surgical procedures and echocardiographic assessment.

In accordance with previously detailed described (40), 2 types of *Lpin1* transgenic male mice and their respective control littermates were subjected to MI, which was induced by the ligation of the left anterior descending coronary artery. To facilitate these experiments, 8- to 10-week-old male mice were anesthetized under isoflurane 2%, followed by intubation and mechanical ventilation. The surgical process involved making a small incision in the left lateral sternum, situated between the fourth and fifth ribs, subsequent to the delicate dissection of overlying skin and muscle layers. Employing a rib retractor, the ribcage was gently expanded to afford access to the LV apex. Ligature sutures were meticulously placed 2–3 mm below an imaginary line connecting the lowest points of the left atrium and LV apex. In contrast, the sham-operated group underwent the same surgical procedure without coronary artery ligation.

For a span of 1 week preceding the surgery and extending to 4 weeks after MI, cardiac function was meticulously monitored through transthoracic echocardiography, using the Vevo2100 ultrasound system (FUJIFILM Visual Sonics, Tokyo, Japan) (Figure 4B). And all mice undergoing MI surgery were checked by echocardiography on the first postoperative day to ensure the success of the surgery and the almost same infarcted degree. On the B-mode parasternal long axis view, heart function was assessed by tracking the endocardium using the provided analysis software (Vevo LAB 5.6.1), meanwhile we obtained end-systolic area and end-diastolic area and FAC. FAC was the difference between the end-diastolic areas (Aread) and the end-systolic areas (Areas) divided by the end-diastolic areas (Aread). FAC was measured as: FAC%= (Aread – Areas) / Aread × 100 (41). It can help to predict outcomes following MI (42). On the B-mode parasternal long axis view, the LVDd was recorded.

One- and 4-weeks following MI surgery, the hearts were examined after a 24-hour fasting period. Inspection under a stereomicroscope revealed that the infarct zone was thin and transparent, but the noninfarct area tended to the normal color of the tissue. Based on these divergent colorations and the position of the ligation, the heart tissue was categorized into 3 distinct zones: a remote zone at the base, a border zone in the middle, and an infarct zone.

### Preparation of tissue sections.

For the acquisition of paraffin sections, 4 weeks after MI surgery, the mice were anesthetized using isoflurane inhalation and sacrificed by cervical dislocation. After thoracic access, a 25-gauge needle was delicately inserted at the cardiac apex to perfuse the heart with cold phosphate-buffered saline (PBS). The heart was then subjected to swift fixation through the deployment of a rapid fixative (Ufix, SAKURA, Tokyo, Japan), a process lasting 24 hours. After fixation, the fixed heart tissues were transitioned to a 75% ethanol solution, where they underwent a 24-hour dehydration cycle. The embedding of these samples was executed utilizing the Cell & Tissue Processor CT-Pro20 (GenoStaff, Tokyo, Japan) and the Tissue Embedding Station EG1160 (Leica Biosystems, Wetzlar, Germany). To ensure uniformity and facilitate future comparative analysis of infarcted area size, each paraffin block was sectioned to the equivalent depth of 400 μm from the ligation site to the apex, and the thickness of each slice was 10 μm.

In the case of frozen sections, 1 week after MI surgery, the mice were similarly anesthetized with isoflurane inhalation and euthanized via cervical dislocation. Perfusion exsanguination with PBS was executed, followed by fixation in the rapid fixative (Ufix) for 24 hours. It’s noteworthy to mention that a subset of samples earmarked for the evaluation of ROS were excluded from Ufix fixation. The fixed hearts were quickly frozen in liquid nitrogen together with Tissue-Tek Optimal Cutting Temperature Compound (4583, SAKURA) and Tissue-Tek Cryomold plastic block (4566, SAKURA). Subsequently, the frozen samples were sectioned to produce 4 μm slices, which were then conserved at a frigid -80°C temperature prior to the staining process.

### H&E and EVG staining.

Four weeks after MI, the excised cardiac specimens underwent a transformation into paraffin sections, subsequently subjected to the process of staining. The objective was to unveil the nuanced histological alterations within the cKO and cOE hearts. The H&E and EVG stained micrographic representations were meticulously captured through the utilization of the All-in-One Fluorescence Microscope BZ-X810 (Keyence, Osaka, Japan). The images were subsequently subjected to rigorous analysis with the aid of the sophisticated BZ-X810 Analyzer software (Keyence, Osaka, Japan).

H&E staining was performed after the deparaffinization and through rinsing with water. The nucleus was stained with Hematoxylin and the cytoplasm was stained with Eosin. EVG staining was performed on infarcted heart specimens after paraffin embedding. This multifaceted staining method targeted the individual delineation of elastic fibers, collagen fibers, and muscle fibers. After deparaffinization and an exhaustive water wash, the samples underwent an immersion phase in fuchsine solution, followed by a fractionation with 100% ethanol. Subsequently, after wash, the cellular constituents were adorned with Weigert’s iron hematoxylin solution. A final step involved immersion in 0.5% hydrochloric acid alcohol, culminating in staining with van Gieson’s solution. The assessment of both the fibrotic regions and the LV free wall areas was meticulously executed through the implementation of ImageJ software (NIH). This sophisticated image analysis facilitated the precise quantification of these specific areas, elucidating the histological modifications induced by the experimental variables.

### LD staining.

One week following the induction of MI, the excised cardiac specimens underwent a meticulous transformation into fixed frozen sections, designated for the specific purpose of LD quantification. The LDs were distinguished by a vivid green fluorescence, named Bodipy 493/503 (D3922, Thermo Fisher Scientific, Waltham, MA, USA), a fluorescent probe custom-tailored for the precise detection of lipid deposits.

Prior to the staining procedure, the slides were put into Ufix for 30 minutes, subsequently followed by a thorough triple wash with PBS. The samples were subjected to an incubation phase within a 4 μM Bodipy solution, The samples were incubated on 4 μM Bodipy solution avoiding light for 30 minutes at room temperature. In addition to the Bodipy stain, Wheat Germ Agglutinin (WGA), Alexa FluorTM555 Conjugate (Thermo Fisher Scientific, Waltham, MA, USA), was used to impart distinction to the cell membranes.

To obviate the impact of autofluorescence originating from cardiac tissue, the Vector TrueVIEW Autofluorescence Quenching Kit with DAPI (SP-8500-15, Vector Laboratories, Newark, CA, USA) was used in this study.

All the Bodipy 493/503 stained images were taken by the Spectral Confocal Microscope LSM 880 (ZEISS, Oberkochen, Germany), further analyzed using the software DP Controller and DP Manager (OLYMPUS). The number of LDs in remote zone, border zone, and infarct zone was calculated by ImageJ.

### IHC staining.

Due to the reason that the anti-CD45 antibody is an indicator of neutrophils, immunostaining of anti-CD45 was performed to detect neutrophils after MI. One week after MI, the harvested hearts were made into fixed frozen sections, which were used for CD45 staining to evaluate the inflammatory damage after MI (Figure 4B). After fixing by Ufix for 30 minutes, the endogenous peroxidases were blocked using 3% H_2_O_2_ (080-01186, FUJIFILM Wako Pure Chemical Corporation) for 10 minutes at room temperature. We used G-Block (GB-01, GenoStaff, Tokyo, Japan) to replace the traditional blocking serum to incubate the slices for 10 minutes at room temperature. Purified Rat Anti-Mouse CD45 (#550539, BD Pharmingen, Frankin Lakes, New Jersey) was used as the primary antibody, which was dissolved in the Normal Goat Serum from the ImmPRESS HRP Goat Anti-Rat IgG, Mouse Adsorbed Polymer Detection Kit, Peroxidase (MP-7444-15, Vector Laboratories). The primary antibody and the slices were incubated overnight at 4°C. After washing with PBS, the second antibody ImmPRESS (Peroxidase) Polymer Anti-Rat IgG from the above kit was incubated with the slices for 30 minutes at room temperature. Then the samples were stained with ImmPACT DAB Substrate Kit, Peroxidase (SK-4105, Vector Laboratories) for about 1 minute. Finally, the slices were stained with hematoxylin and washed with running water for 20 minutes. All the CD45 stained images were taken by the Polarizing Microscope BX51-P (OLYMPUS) and were managed by the software DP Controller and DP Manager (OLYMPUS). The area of CD45^+^ cells in remote zone, border zone, and infarct zone was calculated by ImageJ.

### ROS staining.

To make the staining of ROS, DHE powder (041-28251, FUJIFILM Wako Pure Chemical Corporation, Osaka, Tokyo) was dissolved in Dimethyl Sulfoxide (DMSO, 043-07216, FUJIFILM Wako Pure Chemical Corporation). The resulting solution was then diluted with PBS to a concentration of 10 μM.

The frozen sections were washed with PBS twice and then stained with 10 μM DHE for a duration of 30 minutes at room temperature. This procedure was essential for the quantitative evaluation of ROS levels. Also, the Vector TureVIEW Autofluorescence Quenching Kit with DAPI (SP-8500-15, Vector Laboratories, Newark, CA, USA) was used to effectively bind and quench the autofluorescence of the heart. All the DHE stained images were taken by the Polarizing Microscope BX51-P (OLYMPUS, Tokyo, Japan) and were managed by the software DP Controller and DP Manager (OLYMPUS). Subsequently, the mean gray value of DHE fluorescence was meticulously calculated using ImageJ software (National Institutes of Health, Bethesda, MD, USA), ensuring the reliable quantification of ROS within the analyzed samples.

### H_2_O_2_ analysis.

As previously described (43), 30–50 mg heart powder from the whole frozen heart tissue was used to measure the abundance of H_2_O_2_ by using Amplex Red Hydrogen Peroxide/Peroxidase Assay Kit (A22188, Thermo Fisher Scientific, Waltham, MA, USA). The supernatant derived from the heart homogenate was mixed with 100μM of Amplex Red reagent and 0.2 U/mL of horseradish peroxidase (HRP). The supernatant along with the above mixed working solution was incubated at 37°C for 30 minutes. The absorbance of the resultant solution was determined at 560 nm through the multimode plate reader, namely ARVO X3 (Perkin Elmer, Waltham, MA, USA), and analyzed using the Perkin Elmer 2030 Workstation software (Perkin Elmer, Waltham, MA, USA).

### NAC treatment.

NAC (015-05132, FUJIFILM Wako Pure Chemical Corporation) was dissolved in drinking water at a concentration of 1 mg/mL, and the pH of the solution was adjusted to 7.0. This dose of NAC in drinking water has been reported to exert therapeutic effects in several ROS-related pathologies in mice (44–46). NAC-containing drinking water was administered starting 1 week prior to MI surgery and was continued until the day of sample collection. Drinking water, with or without NAC, was refreshed every 3 days.

### Lipid extraction.

A mixture, comprising chloroform and methanol in a 2:1 ratio, was prepared for the lipid extraction. A total of 20 mg of heart powder derived from the entirety of the frozen cardiac tissue, was mixed with 500 μL of this specialized working solution. After 10 min centrifugation, the supernatant was carefully isolated and then blended with 80 μL distilled water (DW). This step led to the formation of a clear, distinct layering. The upper layer was the water layer, while the lower layer was the chloroform layer. 250 μL solution from the lower layer was extracted and dried in the aluminum block constant temperature bath (MG-3100, EYELA, Tokyo, Japan). Upon completion of the lipid extraction process, the obtained lipids were proficiently dissolved in 50 μL of 2-Propanol (166-04836, FUJIFILM Wako Pure Chemical Corporation) to create the lipid working solution, essential for subsequent analysis.

### Measurement of TAG levels.

The quantification of TAG content was determined utilizing the Triglyceride E-Test kit (432-40201, FUJIFILM Wako Pure Chemical Corporation). Both the samples and the designated standards were combined with a prepared chromogenic reagent, followed by incubation at 37°C for 5 minutes. The fluorometric assay was performed, and the outcomes were recorded using the multimode plate reader, ARVO X3 (Perkin Elmer, Waltham, MA, USA). The gathered data were further analyzed with the specialized Perkin Elmer 2030 Workstation software (Perkin Elmer, USA).

### Measurement of FFA level.

The quantification of FFA levels was determined by the Non-Esterified Fatty Acid C-Test kit (279-75401, FUJIFILM Wako Pure Chemical Corporation). Each sample and standard were mixed with the prepared chromogenic reagent A, followed by an incubation at 37°C for 10 minutes. Then chromogenic reagent B was also prepared and introduced to incubate at 37°C for 10 minutes. The absorbance values were determined at wavelength of 560 nm by the multimode plate reader, ARVO X3 (Perkin Elmer, Waltham, MA, USA), and the software Perkin Elmer 2030 Workstation (Perkin Elmer, USA).

### RNA isolation and quantitative PCR (qPCR).

In the initial phase of experimentation, MI models were induced in WT mice. The expression levels of *Lpin1* were assessed in the remote zone, border zone, and infarct zone one day before surgery, one day after surgery, and 3 days after surgery. In the formal experiment conducted one week after MI, cardiac tissue was harvested and meticulously partitioned into the remote zone, border zone, and infarct zone. To confirm the specificity of transgene occurrence only in the heart, an examination was conducted on both heart tissue and skeletal muscle from *Lpin1* baseline mice. Furthermore, the expression levels of genes related to fatty acid metabolism were measured in *Lpin1* baseline mice.

The extraction of total RNA from heart tissue was accomplished using the RNeasy Fibrous Tissue Mini Kit (74704, QIAGEN, Germantown, MD, USA). Reverse transcription to cDNA was carried out utilizing the reverse transcriptase (ReverTra Ace; TOYOBO, Osaka, Japan). Quantitative reverse transcription-quantitative PCR (RT-qPCR) was performed utilizing QuantStudio5 (Thermo Fisher Scientific, Waltham, MA, USA) with subsequent data analysis conducted through the Thermo Fisher Connect system. The FastStart Essential DNA Green Master (Roche, Basel, Switzerland) was utilized, along with the specified primer sequences presented (Supplemental Table 2). All genetic sequences were comprehensively analyzed.

### Protein extraction and western blot assay.

One week after MI, cardiac tissue was meticulously dissected and categorized into distinct regions—remote zone, border zone, and infarct zone. Similarly, cardiac tissue and skeletal muscle samples from lipin1 baseline mice were scrutinized to validate the exclusive manifestation of the transgene in the heart.

For protein extraction from frozen heart tissue, a homogenization buffer was meticulously prepared following Dr. Schweitzer’s established protocol, comprising 25 mM HEPES, 5 mM EDTA, 150 mM NaCl, 1% Triton X100, pH 8.0 (38). Each 10 mL of the solution was fortified with a 1x PhosStop Phosphatase Inhibitor Cocktail tablet (04906845001, Roche, Basel, Switzerland) and a 1x cOmplete Protease Inhibitor Cocktail EDTA-free tablet (11873580001, Roche, Basel, Switzerland). In accordance with the previous description (47), supernatants were collected post-homogenization with 5 μL/mg of heart tissue homogenization buffer, facilitated by the lysis and homogenization system (Minilys, Bertin Instruments, France).

Heat-denatured protein samples were subjected to SDS-PAGE with 8% acrylamide gels, followed by transfer onto polyvinylidene difluoride (PVDF) membranes (Millipore, Billerica, MA, USA). After blocking with 5% bovine serum albumin (for lipin1) or 1% milk (for actin) for 1 hour at room temperature, the membranes were incubated with the corresponding primary antibody separately overnight at 4°C. Primary antibodies against lipin1 (1:1000; 14906S, Cell Signaling Technology, Danvers, MA, USA) and actin (1:2500; MA5-11869, Thermo Fisher Scientific, Waltham, MA, USA) were used. Secondary antibodies against rabbit IgG (1:10,000; 7074S, Cell Signaling Technology, Danvers, MA, USA) and mouse IgG (1:10,000; GE Healthcare, Little Chalfont, UK) were applied for 3 hours at room temperature. Visualization of protein bands was achieved using the Pierce ECL Prime system (GE Healthcare, Little Chalfont, UK) on the LAS4000 instrument (GE Healthcare, Little Chalfont, UK), with quantification carried out using ImageJ.

### Statistics.

Data are presented as mean ± SEM. Statistical analyses were executed using GraphPad Prism 8 (GraphPad Software). The comparison between 2 groups was conducted through an unpaired 2-tailed *t* test, while multigroup analyses employed ordinary one-way ANOVA followed by Bonferroni’s test to confirm significant differences among groups. The variance of heart function over the 4-week post-MI surgery period was analyzed via 2-way ANOVA. Significance was attributed to *P* < 0.05.

### Study approval.

All animal experiments conducted within this study obtained the requisite approval from the University of Tokyo Ethics Committee for Animal Experiments (approval no. medicine-P20-047) and adhered rigorously to the University of Tokyo Animal Experiment Guidelines, as well as the Animal Research: Reporting of *In Vivo* Experiments (ARRIVE) guidelines. All experiments involving cells and tissues sourced from patients received approval from the IRB of the University of Tokyo Hospital (approval nos. G-10032 and 11801). Patient information is shown in Supplemental Table 1.

### Data availability.

Values for all data points in graphs are available in the Supporting Data Values file.

## Author contributions

JG, HT, KU, and IK conceived and designed the project; JG and KK performed most of the experiments and analyzed data; MK, SN, and KK performed single-nucleus RNA-Seq analysis; M Hashimoto, TK, MI, SB, CZ, RK, HYM, HS, BZ, M Harada, and NT helped in the analysis and interpretation of data; and JG, KK, KU, HT, BNF, and IK wrote the manuscript. All authors read and approved the manuscript.

## Funding support

This work is the result of NIH funding, in whole or in part, and is subject to the NIH Public Access Policy. Through acceptance of this federal funding, the NIH has been given a right to make the work publicly available in PubMed Central.

- Japan Society for the Promotion of Science KAKENHI (21H02908 to KU, 22H00471 to SN, 22K08174 to HT, 21H05045 to IK).
- Japan Agency for Medical Research and Development (AMED) (JP23gm6710012 to KU, JP22ama121016, JP22bm1123011, JP23tm0524009 to SN, JP18km0405209, JP21ek0109569, JP22ek0210172, JP22ek0210167, JP23tm0724607, JP23gm4010020, and JP23tm0524004 to IK).
- Takeda Science Foundation, Mochida Memorial Foundation for Medical and Pharmaceutical Research, Research Grants from Bristol Myers Squibb (to KK).
- Chugai Foundation for Innovative Drug Discovery Science (to KU).
- Generation of Lpin1 transgenic mice supported by R01 HL119225 and P30 DK056341 (to BNF).

## Supplementary Material

Supplemental data

Unedited blot and gel images

Supporting data values

## Version 1 10/30/2025

In-Press Preview

## Version 2 12/08/2025

Electronic publication

## References

[1] TJ CRK K (2017). Heart failure after myocardial infarction in the era of primary percutaneous coronary intervention: mechanisms, incidence and identification of patients at risk. World J Cardiol.

[2] Velagaleti RS (2008). Long-term trends in the incidence of heart failure after myocardial infarction. Circulation.

[3] Bahit MC (2018). Post-myocardial infarction heart failure. JACC Heart Fail.

[4] Jenca D (2021). Heart failure after myocardial infarction: incidence and predictors. ESC Heart Fail.

[5] Cohn JN (2000). Cardiac remodeling--concepts and clinical implications: a consensus paper from an international forum on cardiac remodeling. Behalf of an international forum on cardiac remodeling. J Am Coll Cardiol.

[6] (2000). Left ventricular remodeling after myocardial infarction: pathophysiology and therapy. Circulation.

[7] Heidenreich PA (2022). 2022 AHA/ACC/HFSA guideline for the management of heart failure: a report of the American College of Cardiology/American Heart Association joint committee on clinical practice guidelines. Circulation.

[8] Ponikowski P (2014). Heart failure: preventing disease and death worldwide. ESC Heart Fail.

[9] Machaj F (2019). New therapies for the treatment of heart failure: a summary of recent accomplishments. Ther Clin Risk Manag.

[10] Doenst T (2013). Cardiac metabolism in heart failure: implications beyond ATP production. Circ Res.

[11] Stanley WC (2005). Myocardial substrate metabolism in the normal and failing heart. Physiol Rev.

[12] Shao D, Tian R (2015). Glucose transporters in cardiac metabolism and hypertrophy. Compr Physiol.

[13] Kolwicz Jr SC (2011). Glucose metabolism and cardiac hypertrophy. Cardiovasc Res.

[14] Lopaschuk GD (2021). Cardiac energy metabolism in heart failure. Circ Res.

[15] Langner CA (1989). The fatty liver dystrophy (fld) mutation. A new mutant mouse with a developmental abnormality in triglyceride metabolism and associated tissue-specific defects in lipoprotein lipase and hepatic lipase activities. J Biol Chem.

[16] Péterfy M (2001). Lipodystrophy in the fld mouse results from mutation of a new gene encoding a nuclear protein, lipin. Nat Genet.

[17] Han GS (2006). The Saccharomyces cerevisiae Lipin homolog is a Mg2+-dependent phosphatidate phosphatase enzyme. J Biol Chem.

[18] Sarwar N (2007). Triglycerides and the risk of coronary heart disease: 10,158 incident cases among 262,525 participants in 29 Western prospective studies. Circulation.

[19] Goldberg IJ (2011). Triglycerides and heart disease: still a hypothesis?. Arterioscler Thromb Vasc Biol.

[20] Finck BN (2006). Lipin 1 is an inducible amplifier of the hepatic PGC-1alpha/PPARalpha regulatory pathway. Cell Metab.

[21] Wang W, Ledee D (2021). ACAA2 is a ligand-dependent coactivator for thyroid hormone receptor β1. Biochem Biophys Res Commun.

[22] He L (2012). Carnitine palmitoyltransferase-1b deficiency aggravates pressure overload-induced cardiac hypertrophy caused by lipotoxicity. Circulation.

[23] Kintscher U (2020). The role of adipose triglyceride lipase and cytosolic lipolysis in cardiac function and heart failure. Cell Rep Med.

[24] Stanley WC (2012). Dietary fat and heart failure: moving from lipotoxicity to lipoprotection. Circ Res.

[25] Kelly DP, Strauss SA (1994). Inherited cardiomyopathies. N Engl J Med.

[26] Chambers KT (2021). Myocardial Lipin 1 knockout in mice approximates cardiac effects of human LPIN1 mutations. JCI Insight.

[27] Mitra MS (2011). Cardiac lipin 1 expression is regulated by the peroxisome proliferator activated receptor γ coactivator 1α/estrogen related receptor axis. J Mol Cell Cardiol.

[28] Haemmerle G (2011). ATGL-mediated fat catabolism regulates cardiac mitochondrial function via PPAR-α and PGC-1. Nat Med.

[29] Tikellis C (2008). Cardiac inflammation associated with a Western diet is mediated via activation of RAGE by AGEs. Am J Physiol Endocrinol Metab.

[30] Palomer X (2013). An overview of the crosstalk between inflammatory processes and metabolic dysregulation during diabetic cardiomyopathy. Int J Cardiol.

[31] Zaja I (2014). Cdk1, PKCδ and calcineurin-mediated Drp1 pathway contributes to mitochondrial fission-induced cardiomyocyte death. Biochem Biophys Res Commun.

[32] Ertracht O (2014). The mitochondria as a target for cardioprotection in acute myocardial ischemia. Pharmacol Ther.

[33] Ren J (2010). Mitochondrial biogenesis in the metabolic syndrome and cardiovascular disease. J Mol Med (Berl).

[34] Kim JA (2008). Role of mitochondrial dysfunction in insulin resistance. Circ Res.

[35] Hao Y (2021). Integrated analysis of multimodal single-cell data. Cell.

[36] Fleming SJ (2023). Unsupervised removal of systematic background noise from droplet-based single-cell experiments using CellBender. Nat Methods.

[37] Zhou Y (2019). Metascape provides a biologist-oriented resource for the analysis of systems-level datasets. Nat Commun.

[38] Schweitzer GG (2019). Loss of lipin 1-mediated phosphatidic acid phosphohydrolase activity in muscle leads to skeletal myopathy in mice. FASEB J.

[39] Jama A (2024). Exploring lipin1 as a promising therapeutic target for the treatment of Duchenne muscular dystrophy. J Transl Med.

[40] Konstandin MH (2013). Fibronectin is essential for reparative cardiac progenitor cell response after myocardial infarction. Circ Res.

[41] Benavides-Vallve C (2012). New strategies for echocardiographic evaluation of left ventricular function in a mouse model of long-term myocardial infarction. PLoS One.

[42] DiLorenzo MP (2015). How best to assess right ventricular function by echocardiography. Cardiol Young.

[43] Oka S (2011). PPARα-Sirt1 complex mediates cardiac hypertrophy and failure through suppression of the ERR transcriptional pathway. Cell Metab.

[44] Li C (2024). A novel role for the ROS-ATM-Chk2 axis mediated metabolic and cell cycle reprogramming in the M1 macrophage polarization. Redox Biol.

[45] Li J (2017). BMI-1 mediates estrogen-deficiency-induced bone loss by inhibiting reactive oxygen species accumulation and T cell activation. J Bone Miner Res.

[46] Otte DM (2011). N-acetyl cysteine treatment rescues cognitive deficits induced by mitochondrial dysfunction in G72/G30 transgenic mice. Neuropsychopharmacology.

[47] Ishizuka M (2021). CXCR7 ameliorates myocardial infarction as a β-arrestin-biased receptor. Sci Rep.

