# Supplementary material for: Myocardial lipin1 protects the heart against ischemic injury by preserving lipid homeostasis
Source: JCI Insight. 2025 Oct 30;10(23):e183334. doi: 10.1172/jci.insight.183334 (PMC12890528; doi:10.1172/jci.insight.183334)
Supplement: Supplemental data [file jciinsight-10-183334-s212.pdf]

Supplementary Material

SFig 1

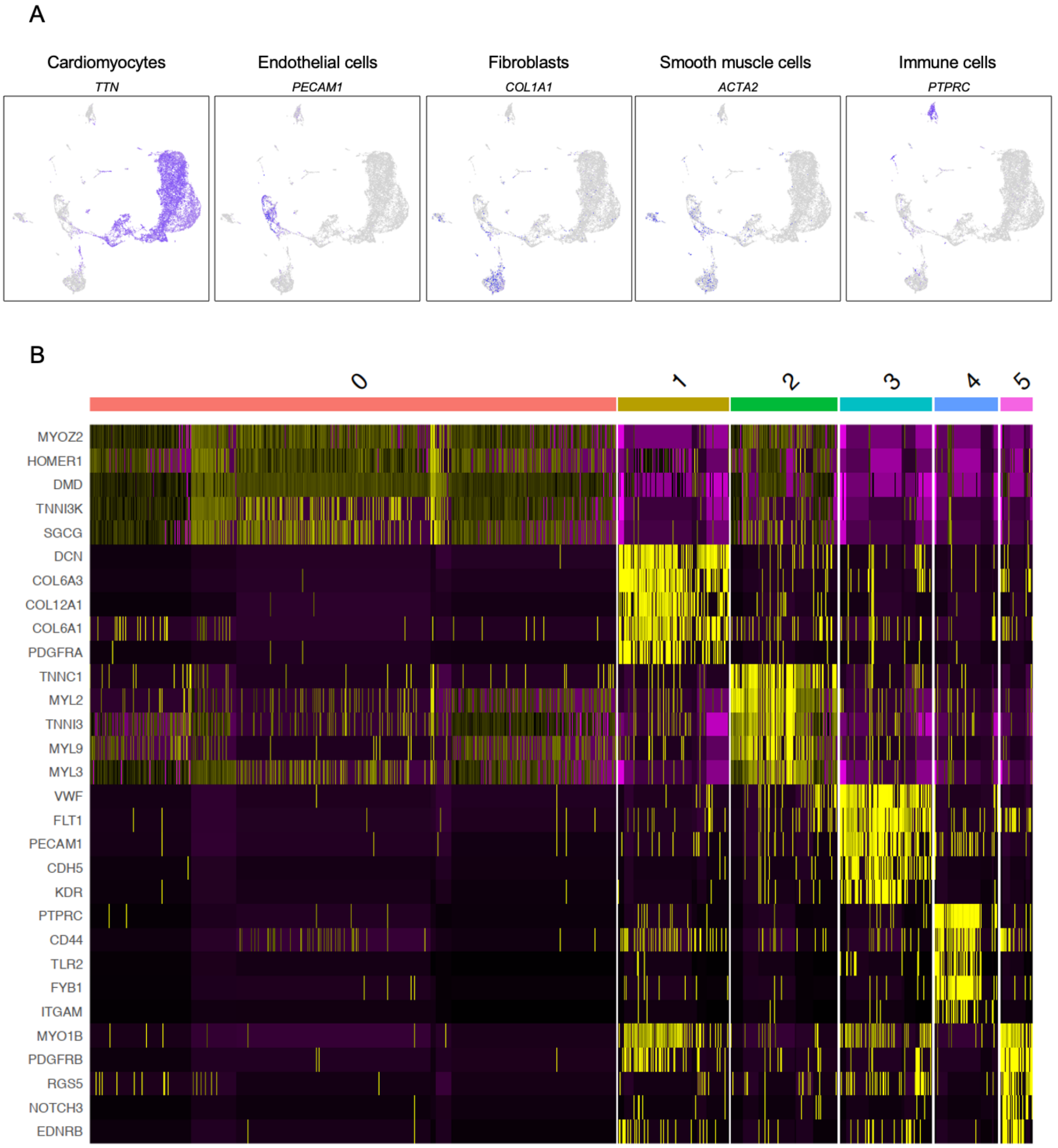

Supplementary Figure 1. Single-cell RNA-seq reveals the expression of genes.

(A) Feature plots of representative genes of each cluster are shown under single-cell analysis. (B) Heatmap indicating the cell-type-specific expression profiles of genes.

SFig 2

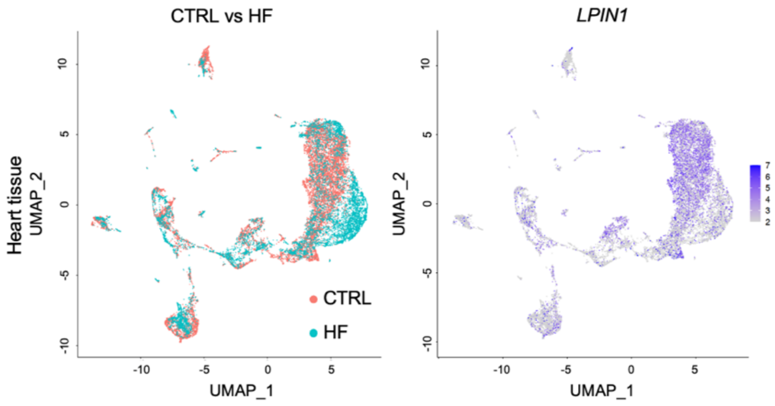

Supplementary Figure 2. Single-cell RNA-seq of heart tissue.

Uniform Manifold Approximation and Projection (UMAP) plot of single-cell transcriptomes of heart tissue cells from three heart failure patients and three control subjects. The cells were color-coded by control or heart failure and *LPIN1* expression is on the UMAP plot.

SFig 3

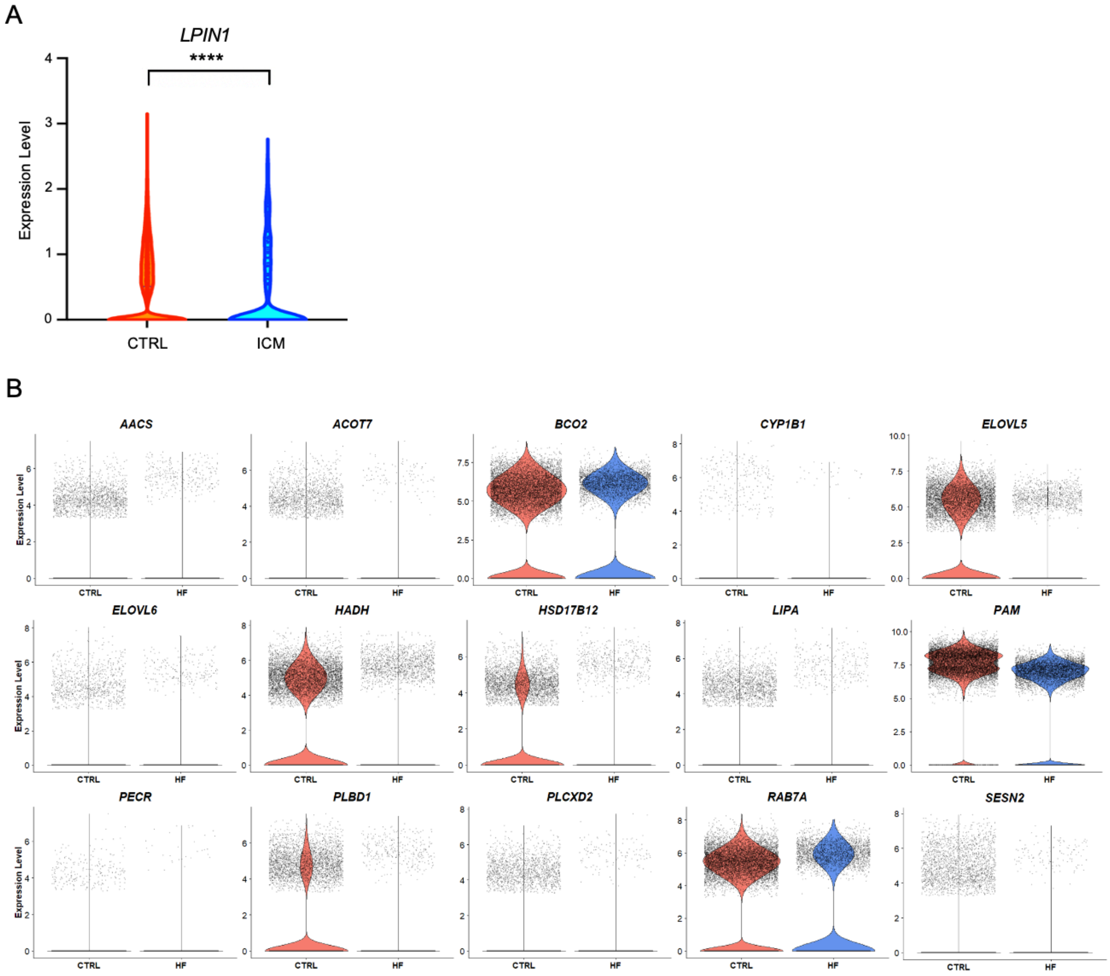

Supplementary Figure 3. Supplemental snRNA-seq data.

(A) The expression level of cardiac LPIN1 was decreased from the ischemic heart failure patient (n=1) compared to the control (n=3). (B) Violin plots showing the expression levels of representative PPARG target genes involved in lipid metabolism in cardiomyocytes, based on snRNA-seq data from non-failing and failing human hearts (n=3 for each). Genes were downregulated in cardiomyocytes from failing hearts, in parallel with reduced LPIN1 expression.

Supplementary Table1

	ID	Sex	Age (y)	LVEF (%)	Cause of Death
Control (n = 3)	1	F	73	72	acute lymphoblastic leukemia
	2	F	52	61	myelodysplastic syndromes
	3	F	57	66	cerebral infarction

	ID	Sex	Age (y)	LVEF (%)	Diagnosis of Heart Disease
Heart Failure (n = 3)	1	M	36	29	dilated cardiomyopathy
	2	M	57	20	ischemic cardiomyopathy
	3	M	28	25	dilated hypertrophic cardiomyopathy

Supplementary Table2

Species	Gene	Sequences	
mouse	*18S rRNA*	forward	5′-CTTAGAGGGACAAGTGGCG-3′
		reverse	5′-ACGCTGAGCCAGTCAGTGTA-3′
mouse	*Lpin1*	forward	5′-CATGCTTCGGAAAGTCCTTCA-3′
		reverse	5′-GGTTATTCTTTGGCGTCAACCT-3′
mouse	*Ppargc1a* （PGC1A）	forward	5′-AAGTGTGGAACTCTCTGGAACTG-3′
		reverse	5′-GGGTTATCTTGGTTGGCTTTATG-3′
mouse	*Pnpla2* （ATGL）	forward	5′-CTGAGAATCACCATTCCCACATC-3′
		reverse	5′-CACAGCATGTAAGGGGGAGA-3′
mouse	*Cpt1b*	forward	5′-CCCATGTGCTCCTACCAGAT-3′
		reverse	5′-CCTTGAAGAAGCGACCTTTG-3′
mouse	*Il1a*	forward	5′-TTGGTTAAATGACCTGCAACA-3′
		reverse	5′-GAGCGCTCACGAACAGTTG-3′
mouse	*Il6*	forward	5′-TAGTCCTTCCTACCCCAATTTCC-3′
		reverse	5′-TTGGTCCTTAGCCACTCCTTC-3′
mouse	*Tnf*	forward	5′-GCCTCTTCTCATTCCTGCTT-3′
		reverse	5′-CTCCTCCACTTGGTGGTTTG-3′
mouse	*Il10*	forward	5′-CTTACTGACTGGCATGAGGATCA-3′
		reverse	5′-GCAGCTCTAGGAGCATGTGG-3′
