# Supplementary material for: Myocardial lipin1 protects the heart against ischemic injury by preserving lipid homeostasis
Source: JCI Insight. 2025 Oct 30;10(23):e183334. doi: 10.1172/jci.insight.183334 (PMC12890528; doi:10.1172/jci.insight.183334)

Full unedited gel for Figure 2B Lipin1 and β-actin

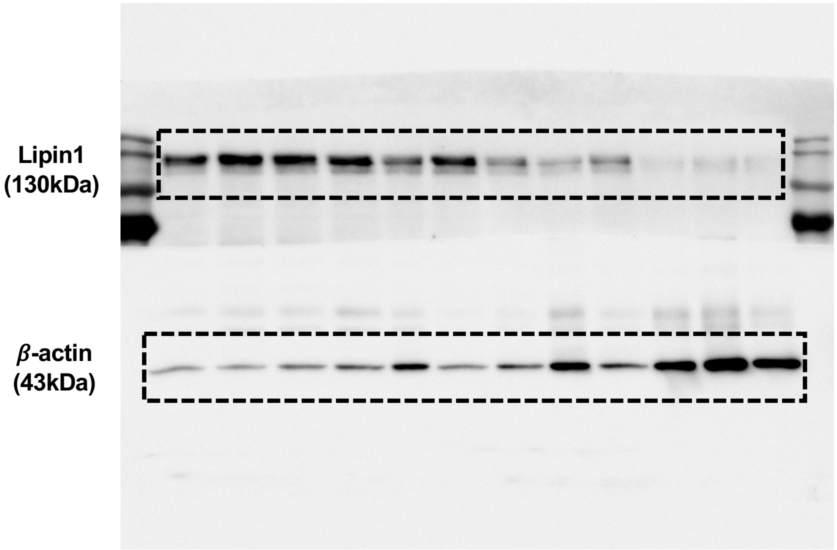

Full unedited gel for Figure 2D Lipin1and β-actin (cKO)

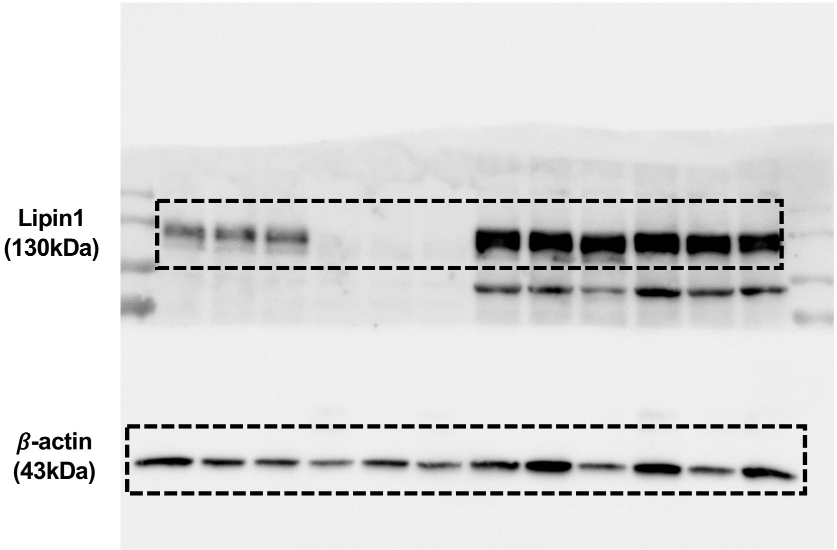

Full unedited gel for Figure 2D Lipin1and β-actin (cOE)

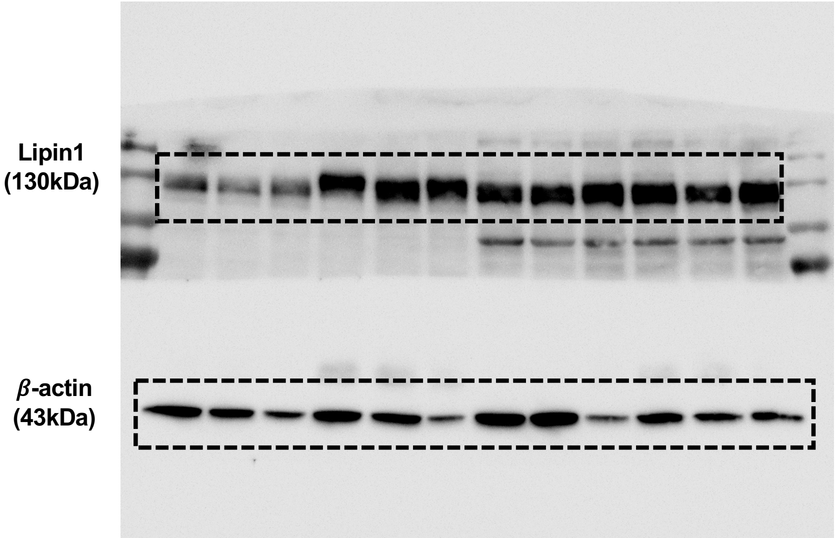

Supplement: Unedited blot and gel images [file jciinsight-10-183334-s213.pdf]
